# Salivary Huntingtin protein is uniquely associated with clinical features of Huntington’s disease

**DOI:** 10.1038/s41598-023-28019-y

**Published:** 2023-01-19

**Authors:** Georgia M. Parkin, Jody Corey-Bloom, Chase Snell, Haileigh Smith, Angela Laurenza, Manuel Daldin, Alberto Bresciani, Elizabeth A. Thomas

**Affiliations:** 1 Department of Epidemiology, University of California Irvine, Irvine, CA USA; 2 Institute for Interdisciplinary Salivary Bioscience Research, University of California Irvine, Irvine, CA USA; 3 Department of Neurosciences, University of California San Diego, San Diego, CA USA; 4 Department of Translational Biology, IRBM S.p.A., via Pontina Km 30, 600, Pomezia, Rome, Italy; 5 Present Address: Menarini Ricerche S.p.A., via Tito Speri 10, Pomezia, Rome, Italy; 6 Present Address: Exscientia, Oxford Science Park, Oxford, UK

**Keywords:** Biomarkers, Huntington's disease

## Abstract

Measuring Huntingtin (HTT) protein in peripheral cells represents an essential step in biomarker discovery for Huntington’s Disease (HD), however to date, investigations into the salivary expression of HTT has been lacking. In the current study, we quantified total HTT (tHTT) and mutant HTT (mHTT) protein in matched blood and saliva samples using single molecule counting (SMC) immunoassays: 2B7-D7F7 (tHTT) and 2B7-MW1 (mHTT). Matched samples, and clinical data, were collected from 95 subjects: n = 19 manifest HD, n = 34 premanifest HD (PM), and n = 42 normal controls (NC). Total HTT and mHTT levels were not correlated in blood and saliva. Plasma tHTT was significantly associated with age, and participant sex; whereas salivary mHTT was significantly correlated with age, CAG repeat length and CAP score. Plasma and salivary tHTT did not differ across cohorts. Salivary and plasma mHTT were significantly increased in PM compared to NC; salivary mHTT was also significantly increased in HD compared to NC. Only salivary tHTT and mHTT were significantly correlated with clinical measures. Salivary HTT is uniquely associated with clinical measures of HD and offers significant promise as a relevant, non-invasive HD biomarker. Its use could be immediately implemented into both translational and clinical research applications.

## Introduction

Huntington’s Disease (HD) is a progressive, genetic neurodegenerative disorder caused by unstable CAG repeat expansions in the first exon of the Huntingtin gene (*HTT).* This mutation translates into a polyglutamine repeat in the Huntingtin protein, the length of which varies by CAG repeat expansion number. Pathogenesis in HD arises largely from the expression of the mutant huntingtin protein (mHTT), leading to the formation of toxic soluble protein oligomers and insoluble aggregates that contribute to the disruption of many intracellular, predominantly cortical and striatal pathways. The pathological expression of mHTT leads to the progressive development of a range of clinical signs and symptoms, which may begin with subtle cognitive and behavioral changes followed by the onset of involuntary motor symptoms, ultimately leading to a premature death 15–20 years after onset^1–4^. Conversely, full-length huntingtin protein has proposed roles in neurogenesis, axonal transport and synaptic transmission, as well as in preventing apoptosis and excitotoxicity^5^. Previous studies have reported associations between cerebrospinal (CSF) mHTT and functional, cognitive and motor symptom progression^6–8^, and we have previously reported an association between salivary total huntingtin protein (tHTT) levels and disease progression^9^. To our knowledge, no study has comprehensively analyzed salivary mHTT in HD, or compared salivary tHTT and mHTT to levels in plasma. The aim of this study was therefore to quantify levels of tHTT and mHTT in saliva and plasma of premanifest and manifest HD patients, as well as normal controls, and investigate associations between these markers with clinical measures.

## Methods

### Human subjects

This study was approved by the University of California, San Diego (UCSD) Institutional Review Board, in accordance with the requirements of the Code of Federal Regulations on the Protection of Human Subjects, and conducted in accordance with the Declaration of Helsinki. Patients were recruited from the UCSD Huntington’s Disease Society of America (HDSA) Center of Excellence, and carried a genetic confirmation of the *HTT* mutation with family history of the disorder. Premanifest (PM) HD individuals had more than ≥ 38 CAG repeats, and a Unified Huntington's Disease Rating Scale (UHDRS) diagnostic confidence rating below 4. Manifest HD patients had a diagnostic confidence rating of 4, indicating that a clinician had ≥ 99% certainty that the patient presented with “‘unequivocal presence of an otherwise unexplained extrapyramidal movement disorder”^10^. The UHDRS was developed by the Huntington Study Group, and is used as a major outcome measure in controlled clinical trials^10,11^. Normal controls (NC) had no reported history of neurological conditions, psychiatric disorders or gout, and no use of psychoactive substances or medications. All participants gave written informed consent prior to sample collection. Demographic and disease data were collected at the time of sample collection, including sex, age, CAG repeat length, and years of education.

### Clinical assessment

PM and HD study participants underwent a single clinical assessment, which included cognitive testing, behavioral and functional measures, and motor ratings. The cognitive battery included the Mini-Mental State Examination (MMSE)^12^, Montreal Cognitive Assessment (MoCA)^13^, Symbol Digit Modalities test (SDMT)^14^ and Stroop word reading test (SWR). Behavioral and psychiatric changes were assessed using the short form Problem Behaviors Assessment (PBA-s)^15^ and the Hospital Anxiety and Depression Scale/Snaith Irritability Scale (HADS-SIS)^16^. Functional proficiency was evaluated using the UHDRS^10^ Total Functional Capacity (TFC). Motor dysfunction was assessed using the UHDRS Total Motor Score (TMS). The sum of all maximal chorea sub-scores were also noted^10^. The SWR, SDMT, TFC and TMS were also incorporated into the composite UHDRS score (cUHDRS) as an additional measure of disease progression^17^. Predicted years to 60% probability of manifest disease onset was calculated using a formula which incorporate CAG repeat length and current age, determined by Langbehn and colleagues^18^. The normalized prognostic index (PIN) score for predicting PM disease progression^19^, which incorporates patient CAG repeat length, age, SDMT score, and TMS^19^ was also calculated, as an additional measure of disease progression.

### Sample collection

Blood from consenting individuals was drawn by venipuncture into 2 ml lavender/EDTA tubes. EDTA/whole blood was mixed well by inversion and spun at 900 g for 15 min. The supernatant was isolated, aliquoted into 1 ml aliquots, snap frozen and stored at − 80 °C. All donors were asked to refrain from smoking, eating, drinking, or oral hygiene procedures for at least 1 h prior to sample collection. Saliva samples were collected between 10 a.m. and 4 p.m. using the passive drool method according to previously established protocols^20^. Roughly 2 ml of unstimulated whole saliva was obtained. Samples were immediately frozen at − 20 °C at the time of collection, then stored at − 80 °C. At the time of use, saliva samples were thawed and centrifuged (10,000×*g*; 10 min; 4 °C) to remove insoluble material and cellular debris. All blood and saliva samples were kept cold prior to the running of assays, to reduce the likelihood of protein degradation.

### Biomarker protein analysis

Levels of tHTT and mHTT protein in saliva and plasma were analyzed at IRBM S.p.A, Italy. Prior to analysis, plasma samples were processed by centrifugation (14000 rpm × 10 min at 4 °C) to remove debrides, and absolute Tween-20 was added to the resulting supernatant. Saliva samples were diluted 1:1 in 2× Lysis buffer (2× phosphate buffered saline, 0.8% Triton-X, protease inhibitor) in order to avoid proteolysis from digestive enzymes. Then samples were then clarified from debrides by centrifugation (14000 rpm × 10 min at 4 °C). Total HTT and mHTT was measured in each biofluid using the 2B7-D7F7 Single Molecule Counting (SMC) assay and three technical replicates^21^ for tHTT, mHTT was measured using the 2B7-MW1 SMC assay and two technical replicatesHTT^22^, on an Erenna^®^ platform. The 2B7-MW1 SMC assay is specific for mHTT, whereas the 2B7-D7F7 assay quantifies HTT protein in a polyglutamine length-independent manner (mHTT and non-expanded wild-type HTT combined)^21,22^. Plasma samples were analyzed using 15 μL/well for 2B7-D7F7 SMC assay and 135 μL/well for the 2B7-MW1 SMC assay. Saliva samples were analyzed using 150 μL/well for 2B7-D7F7 SMC assay and for 2B7-MW1 SMC assay^22^. A calculated concentration above the lower limit of quantification (LLoQ) was obtained for 99% of tHTT and 54% mHTT in plasma, and 95% tHTT and 62% mHTT in saliva. Most values below LLoQ belonged to the control cohort (Table 1). Values below the lower limit of quantification (LLoQ) were substituted with 2.5 fM (half LLoQ) for analysis.

**Table 1:** Range of salivary and plasma total and mutant HTT values.

Measure	Cohort n(NC) = 42 n(gene(+)) = 53	Median, range	% of values below LLOQ (below/above)
Plasma total HTT [fM]	NC	515.3, 198.9–1837.0	0% (0/42)
	Gene (+)	474.8, 144.0–6580.0	2% (1/52)
Plasma mutant HTT [fM]	NC	11.8, 0.8–861.1	43% (18/24)
	Gene (+)	55.2, 1.2–1220.0	28% (12/41)
Salivary total HTT [fM]	NC	851.0, 11.1–6308.0	7% (3/38)
	Gene (+)	374.2, 8.6–4004.0	2% (1/50)
Salivary mutant HTT [fM]	NC	4.5, 2.0–247.0	34% (17/23)
	Gene (+)	43.1, 2.3–1381.0	16% (8/43)

Gene (+) participants are those with the *HTT* gene mutation and include PM and HD. Three saliva samples (n = 1 NC, n = 1 PM, n = 1 HD) were used for preliminary testing, and not including in analyses.

### Western blot analysis

Total protein levels were measured a DC Protein Assay (Bio-Rad Laboratories, Hercules, CA, USA) on the day of and prior to gel electrophoresis. Samples were diluted to 800 pg/ml with deionized water, and then mixed 3:4 with NuPAGE™ LDS Sample Buffer (4×) (Thermo Fisher Scientific, MA, USA). The samples were denatured for 10 min at 70 °C, and 15 µg total protein was subsequently loaded into wells of a NuPAGE™ 3 to 8%, Tris–Acetate, 1.5 mm, Mini Protein Gel (Thermo Fisher Scientific), Proteins were separated at 150 V with NuPAGE™ 1 × Tris–Acetate Running Buffer until the sample buffer had migrated to 1 cm from the bottom of the gel (approximately 1 h). The proteins were then transferred to a Thermo Fisher nitrocellulose membrane at a constant 40 mA overnight with icepack cooling and NuPAGE™ 1× LDS Transfer Buffer. The nitrocellulose membranes were probed with Ponceau S staining (Cell Signalling Technology, Frankfurt, Germany), blocked for 1 h in Tris buffered saline with 20% Tween-20 (Thermo Fisher Scientific) and 5% non-fat milk powder (5% NFMP TBST). The nitrocellulose membranes were then probed via overnight incubation with either 1:500 anti-total HTT antibody (4E10, Santa Cruz Biotechnology, Inc., CA, USA) or anti-mutant HTT antibody (MW1, Developmental Studies Hybridoma Bank, IA, USA) in 5% NFMP TBST, followed by 1:10,000 goat anti-mouse IgG:HRP-conjugated antibody (Merck, NY, USA) in 5% NFMP TBST for 1 h. 4E10 and MW1 antibody are well characterized for tHTT and mHTT, respectively^23–25^. Bands were detected with Pierce™ ECL Western Blotting Substrate, imaged for 5 min (4E10) or 8 min (MW1) on a FluoroChem M Imaging system (Bio-techne, MN, USA). Molecular weight markers were determined using the PageRuler™ Unstained Protein Ladder, with bands marked in pencil following Ponceau S staining, and imaged using the white-light function on the FluoroChem M Imaging system.

### Statistical analysis

Analyses were conducted with GraphPad Prism version 8.4.2 for Windows (GraphPad Software, La Jolla, CA, USA). Twelve matched saliva and blood samples from *HTT-*mutation carriers (13% of total cohort) were collected and analyzed separately as part of a unique, second batch (batch 2); significant batch variation was accounted for through global linear normalization of these samples, using the ratio of the batch 1 *HTT-*mutation carrier sample mean divided by the batch 2 sample mean, for each analyte (Suppl. Fig. 1)^26,27^. Overall, three participants had both salivary tHTT and mHTT below the lower limit of detection, and one participant had both plasma tHTT and mHTT below the lower limit of detection; these values were excluded. One additional salivary tHTT value and one plasma tHTT value were identified visually as outliers and excluded (Suppl Fig. 2). Analyses which corrected for covariates were conducted using IBM^®^ SPSS^®^ Statistics version 25 for Windows (IBM Corp., NY, USA). As unquantified values below LLoQ were substituted with a value of 2.5 fM, all comparison which used continuous data were tested using non-parametric analyses. Mutant HTT values were log-transformed for graphical presentation, after statistical testing. The comparison of plasma and salivary tHTT levels by diagnostic group was conducted using Mann–Whitney U-test, followed by Quade’s non-parametric analysis of covariance (Quade’s ANCOVA), correcting for covariates. Due to the high proportion of LLoQ-substituted mHTT values, particularly in the control cohort, comparisons between diagnostic cohorts for mHTT were conducted using Fisher’s exact test for values over and below LLoQ. All tests conducted were two-sided.

## Results

### Participant characteristics

Plasma and saliva were collected from 95 individuals: 42 NC, 34 PM and 19 HD. Demographic characteristics are summarized in Table 2. Cohorts differed significantly by age (Kruskal–Wallis χ^2^ = 10.86, *p* = 0.004); the PM cohort was significantly younger than the NC cohort (*p* = 0.003). HD patients had significantly higher CAP scores (*p* = 0.0001) compared to the PM cohort (Table 2). Plasma tHTT (r = 0.29, *p* = 0.005), and salivary mHTT (r = − 0.27, *p* = 0.01) but not plasma mHTT or salivary tHTT, was significantly correlated with age, irrespective of diagnostic cohort. Plasma tHTT, but no other HTT measure, was moderately correlated with years of education (r = 0.20, *p* = 0.05). Salivary mHTT, but no other HTT measure, was significantly correlated with CAG repeat length (r = 0.33, *p* = 0.02) and CAP score (r = 0.34, *p* = 0.01). Plasma tHTT (U = 723, *p* = 0.005), but no other measure, differed significantly by participant sex.

**Table 2:** Demographic and disease data for subjects used in this study (median, range).

	NC (n = 42)	PM (n = 34)	HD (n = 19)	*p*
Sex (% male)	52%	62%	53%	0.68
Age [years]	57.0, 25.0–71.0	43.5, 24.0–62.0	49.0, 33.0–65.0	**0.003** (NC vs PM)
Education [years]	16.0, 12.0–20.0	16.0, 12.0–20.0	14.0, 5.0–20.0	0.13
CAG repeat	–	42.0, 39.0–45.0	43.0, 40.0–47.0	0.09
CAP score	–	365.6, 176.2–517.1	457.7, 330.3–627.0	**0.0001**

Statistical tests used: Sex, Chi square test; Age, Education: Kruskal–Wallis test; CAG repeat: Mann–Whitney U test; CAP score: t-test.

CAP, CAG-Age Product score; HD, Huntington’s Disease; NC, normal control; PM, pre-manifest.

Significant values are in bold.

### Diagnostic potential of plasma and salivary tHTT and mHTT

**Figure 1:**
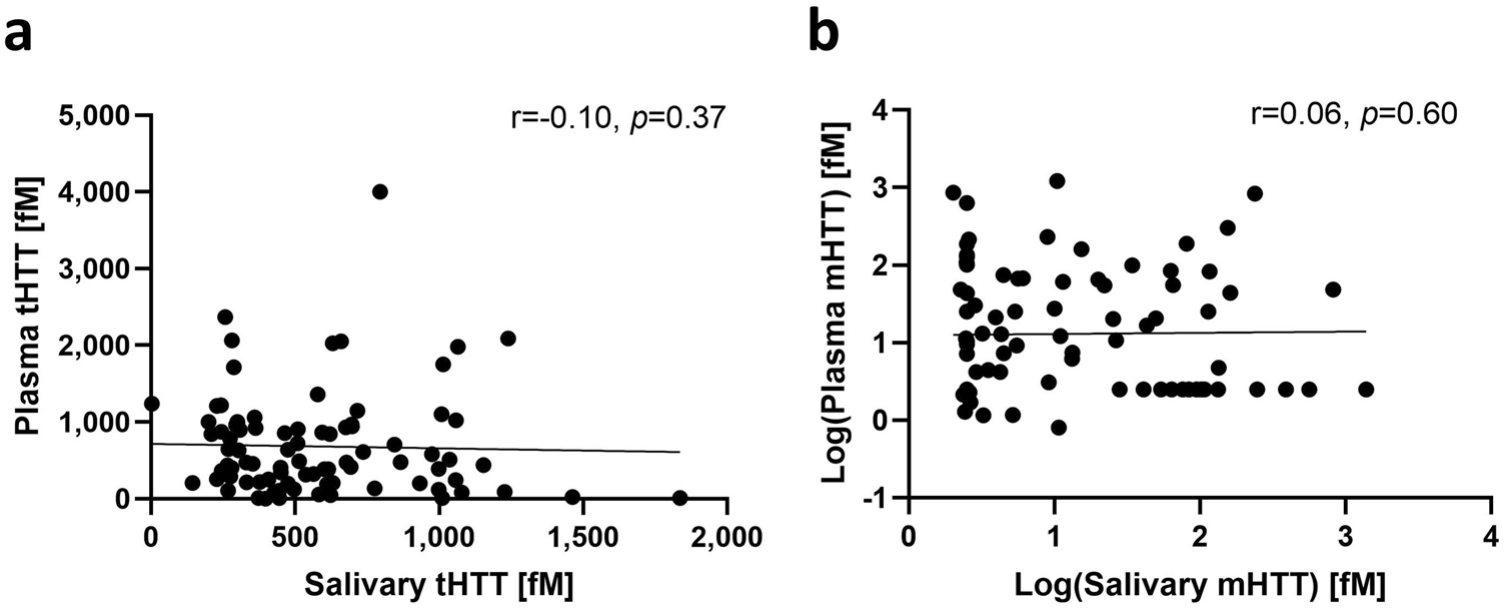
Associations between plasma and salivary tHTT (**a**) and mHTT (**b**) in *HTT* mutation-carriers. Data in (**b**) was log-transformed for graphical presentation after statistical testing. Statistical comparisons conducted using Spearman’s rho.

Total HTT (Fig. 1a) and mHTT levels (Fig. 1b) were not correlated in saliva and plasma.

**Figure 2:**
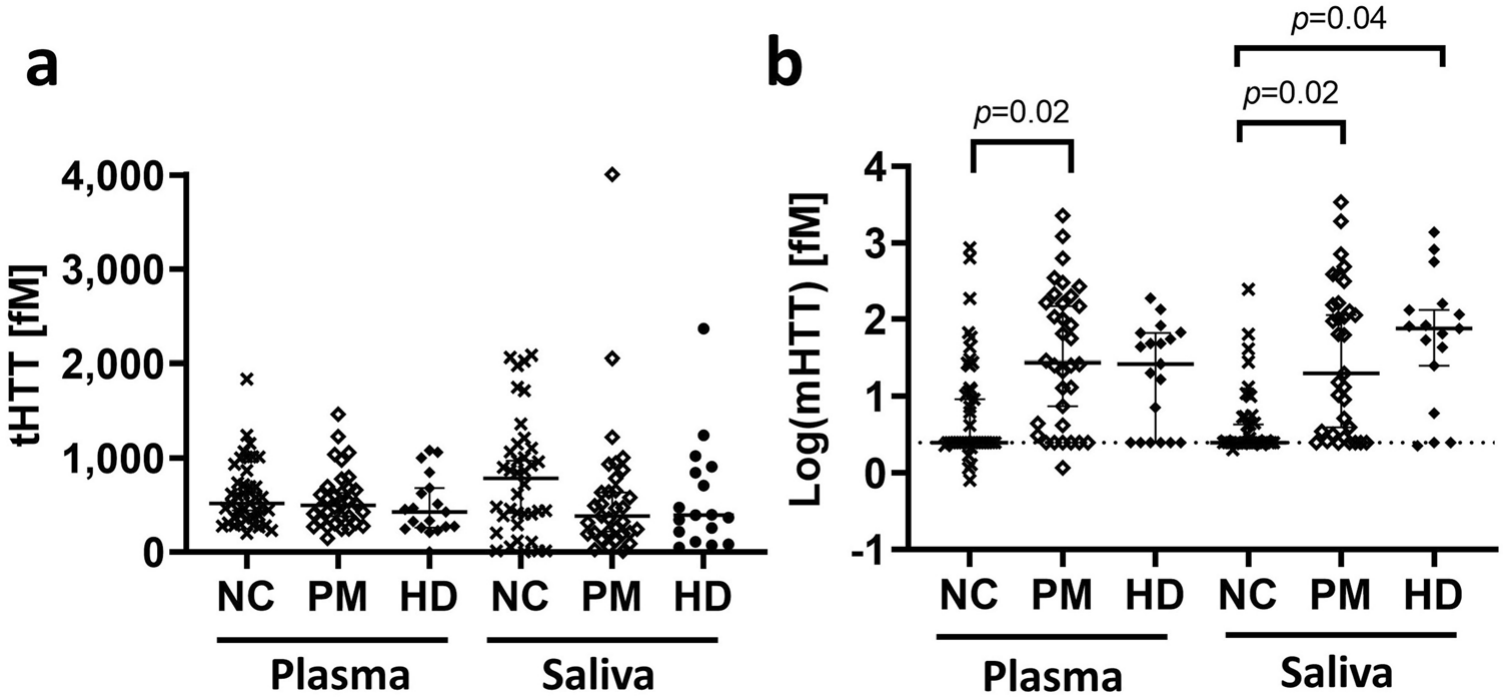
Total HTT (**a**) and mutant HTT (**b**) levels in saliva and plasma of normal controls (NC), premanifest HD individuals (PM) and manifest HD patients (HD). Plasma and salivary tHTT did not differ by diagnostic cohort (**a**). Plasma mHTT was more frequently below the Lower Limit of Quantification (LLoQ) in NC compared to PM, and salivary mHTT levels were more frequently below LLoQ in NC compared to HD and PM (**b**). Total HTT significance determined by Kruskal–Wallis test; mHTT significance determined by Fisher’s exact test. Data in (**b**) was log-transformed for graphical presentation after statistical testing; *p < 0.05.

Plasma tHTT and salivary tHTT were not significantly different across diagnostic groups (> 0.05) (Fig. 2a). The number of plasma tHTT values below LLOQ across different diagnostic cohorts was just above statistical significance (χ^2^ = 5.70, *p* = 0.058); posthoc chi-square tests showed that there were significantly more plasma mHTT values below LLOQ in the NC cohort compared to PM (χ^2^ = 5.53, *p* = 0.02; OR, 95% CI = 3.5, 1.2–10.5) but no difference between NC and HD, or PM and HD (p > 0.05) (Fig. 2b). The number of salivary mHTT values below LLOQ were significantly different across diagnostic cohorts (χ^2^ = 7.54, *p* = 0.02); posthoc chi-square tests showed that there were significantly more salivary mHTT values below LLOQ in the NC cohort, compared to PM (χ^2^ = 5.16, p = 0.02, OR, 95% CI = 3.7, 1.2–10.2), and compared to HD (χ^2^ = 4.22, *p* = 0.04; OR, 95% CI = 4.9, 1.1–23.5), but no difference between PM and HD cohorts (*p* > 0.05) (Fig. 2b).

### Correlations between plasma and saliva tHTT and mHTT, and clinical symptoms in HTT mutation-carriers

Correlations were analyzed using the PM, HD, and combined PM + HD (gene (+)) cohort, both without (Table 3) and with adjusting for age, CAG repeat length, and sex (Suppl. Table 1). In the PM cohort, salivary tHTT and mHTT were significantly correlated with each other, TMS, Chorea, and DCL scores. In addition, salivary mHTT was also correlated with Timed Up and Go (TUG) score. In the HD cohort, salivary tHTT was associated with TMS and Chorea scores, and salivary mHTT was associated with age and CAG repeat number. In the combined *HTT*-mutation carrier cohort (PM + HD), salivary mHTT levels were associated with salivary tHTT levels, CAG repeat number, CAP score, PIN score, predicted years to 60% probability of manifest symptom onset, Independence score, TMS, Chorea, DCL, and TUG (Table 3). Similar significant correlations were observed after correcting for age, CAG repeat number, and sex (Suppl. Table 1). Associations between salivary mHTT and salivary tHTT, and TMS, in the PM cohort, and CAP score, predicted years to 60% manifest symptom onset, TMS, DCL in the PM + HD cohort, passed Bonferrori-adjusted p value cut-off scores (Table 3). Significant correlations are presented graphically in Suppl. Fig. 3 (salivary tHTT), and Suppl. Fig. 4 (salivary mHTT).

**Table 3:** Relationship between plasma and salivary tHTT, mHTT, with demographic and clinical factors (r, p).

	PM (n = 34)				HD (n = 19)				PM + HD (n = 53)			
	Plasma tHTT	Plasma mHTT	Salivary tHTT	Salivary mHTT	Plasma tHTT	Plasma mHTT	Salivary tHTT	Salivary mHTT	Plasma tHTT	Plasma mHTT	Salivary tHTT	Salivary mHTT
Plasma tHTT		0.22, 0.22	− 0.18, 0.34	− 0.05, 0.78		− 0.01, 0.95	− 0.18, 0.48	− 0.26, 0.32		0.14, 0.34	− 0.16, 0.27	− 0.16, 0.29
Plasma mHTT			− 0.23, 0.19	− 0.09, 0.63			0.19, 0.48	− 0.31, 0.24			− 0.11, 0.46	− 0.22, 0.13
Salivary tHTT				**0.59, < 0.001**				0.35, 0.18				**0.48, < 0.001**
Demographic data												
Age	0.16, 0.37	− 0.02, 0.90	0.08, 0.64	0.14, 0.43	0.06, 0.81	0.20, 0.42	− 0.25, 0.34	**− 0.60, 0.01**	0.05, 0.72	− 0.02, 0.91	− 0.02, 0.90	− 0.03, 0.84
Education	**0.35, 0.047**	− 0.03, 0.87	0.02, 0.90	− 0.20, 0.28	0.12, 0.65	− 0.12, 0.64	0.39, 0.12	0.35, 0.17	0.21, 0.14	− 0.01, 0.96	0.14, 0.32	− 0.07, 0.64
Disease data												
CAG	0.06, 0.76	0.12, 0.52	− 0.16, 0.36	0.12, 0.51	− 0.08, 0.74	0.02, 0.94	0.15, 0.58	**0.57, 0.02**	− 0.04, 0.77	0.04, 0.80	− 0.05, 0.74	**0.33, 0.02**
CAP	0.10, 0.57	0.03, 0.85	0.04, 0.84	0.31, 0.08	− 0.16, 0.52	0.19, 0.44	− 0.27, 0.29	0.17, 0.51	− 0.11, 0.44	0.00, 0.99	− 0.03, 0.83	**0.34, 0.01**
PIN score	0.19, 0.30	0.22, 0.21	0.08, 0.64	0.31, 0.08	− 0.13, 0.59	− 0.08, 0.74	− 0.46, 0.06	0.06, 0.82	− 0.08, 0.57	0.03, 0.83	− 0.05, 0.75	**0.34, 0.02**
YTO 60%	− 0.11, 0.56	− 0.03, 0.88	− 0.05, 0.80	− 0.32, 0.07	0.16, 0.50	− 0.14, 0.58	0.24, 0.36	− 0.18, 0.48	0.11, 0.46	0.02, 0.88	0.01, 0.96	**− 0.37, 0.009**
Clinical data												
TFC	− 0.10, 0.57	− 0.25, 0.15	− 0.14, 0.45	− 0.14, 0.45	0.42, 0.08	− 0.08, 0.74	0.37, 0.14	− 0.25, 0.34	0.17, 0.23	− 0.16, 0.26	0.03, 0.86	− 0.26, 0.07
Independence	− 0.27, 0.12	− 0.31, 0.07	− 0.09, 0.61	− 0.22, 0.23	0.25, 0.30	− 0.10, 0.68	− 0.06, 0.83	− 0.37, 0.14	0.05, 0.73	− 0.19, 0.17	− 0.08, 0.60	**− 0.28, 0.049**
SDMT	− 0.24, 0.19	− 0.24, 0.17	− 0.02, 0.90	− 0.08, 0.65	0.18, 0.46	0.14, 0.56	0.26, 0.31	− 0.14, 0.59	0.06, 0.69	− 0.09, 0.52	0.05, 0.73	− 0.19, 0.18
MoCA	− 0.14, 0.42	− 0.02, 0.93	0.04, 0.81	− 0.15, 0.40	0.06, 0.82	0.10, 0.67	0.24, 0.35	− 0.04, 0.89	− 0.04, 0.80	0.05, 0.71	0.09, 0.52	− 0.13, 0.38
MMSE	0.07, 0.71	0.12, 0.49	− 0.11, 0.53	− 0.18, 0.31	− 0.01, 0.98	0.19, 0.44	0.33, 0.20	0.02, 0.94	0.09, 0.53	0.14, 0.31	0.03, 0.85	− 0.23, 0.11
TMS	− 0.04, 0.83	0.20, 0.26	**0.37, 0.04**	**0.52, 0.002**	0.00, 0.99	− 0.24, 0.31	**− 0.59, 0.01**	− 0.03, 0.92	− 0.13, 0.36	− 0.01, 0.92	0.11, 0.47	**0.43, 0.002**
Chorea	− 0.11, 0.54	− 0.15, 0.38	**0.44, 0.01**	**0.40, 0.02**	0.14, 0.56	− 0.19, 0.43	**− 0.54, 0.03**	− 0.34, 0.19	− 0.16, 0.27	− 0.17, 0.22	0.13, 0.36	**0.33, 0.02**
DCL	− 0.12, 0.51	0.12, 0.49	**0.38, 0.03**	**0.47, 0.005**	**–**	**–**	**–**	**–**	− 0.15, 0.28	− 0.01, 0.94	0.20, 0.17	**0.42, 0.002**
PBA total	0.14, 0.43	− 0.05, 0.80	0.26, 0.15	0.17, 0.34	− 0.20, 0.41	0.22, 0.37	0.46, 0.06	0.06, 0.83	− 0.01, 0.95	0.04, 0.78	**0.32, 0.02**	0.20, 0.17
HADSSIS-total	0.12, 0.51	− 0.09, 0.63	0.22, 0.21	0.06, 0.72	− 0.32, 0.18	0.38, 0.11	0.24, 0.35	0.09, 0.74	− 0.08, 0.60	0.07, 0.60	0.23, 0.11	0.13, 0.36
TUG	0.17, 0.46	− 0.05, 0.82	0.22, 0.33	**0.44, 0.04**	0.11, 0.68	− 0.09, 0.74	− 0.12, 0.66	0.31, 0.27	0.09, 0.59	− 0.08, 0.61	0.08, 0.64	**0.45, 0.006**
SWR	− 0.15, 0.44	− 0.19, 0.32	− 0.11, 0.56	− 0.25, 0.18	0.22, 0.36	0.31, 0.20	0.10, 0.70	− 0.10, 0.71	0.01, 0.95	− 0.03, 0.83	− 0.05, 0.76	− 0.19, 0.20
cUHDRS	− 0.19, 0.31	− 0.22, 0.25	− 0.20, 0.28	− 0.26, 0.17	0.26, 0.29	0.14, 0.57	0.35, 0.17	0.35, 0.17	0.05, 0.74	− 0.10, 0.47	− 0.04, 0.82	− 0.22, 0.13

CAP, CAG-Age Product; YTO 60%, years to predicted manifest onset at 60% probability; TFC, Total Functional Capacity; SDMT, Symbol Digit Modalities Test; MoCA, Montreal Cognitive Assessment; MMSE, Mini-Mental State Examination; TMS, Total Motor Score; DCL, Diagnostic Confidence Interval; PBA Total, PBA, Problem Behaviors Assessment Total, HADS-SIS, Hospital Anxiety and Depression Scale-Snaith's Irritability Scale; TUG, Timed Up and Go; cUHDRS, composite Huntington’s Disease Rating Scale.

Bold cells were significant (*p* < 0.05) before adjusting for covariates.

### Total HTT protein isoforms vary in saliva and plasma

**Figure 3:**
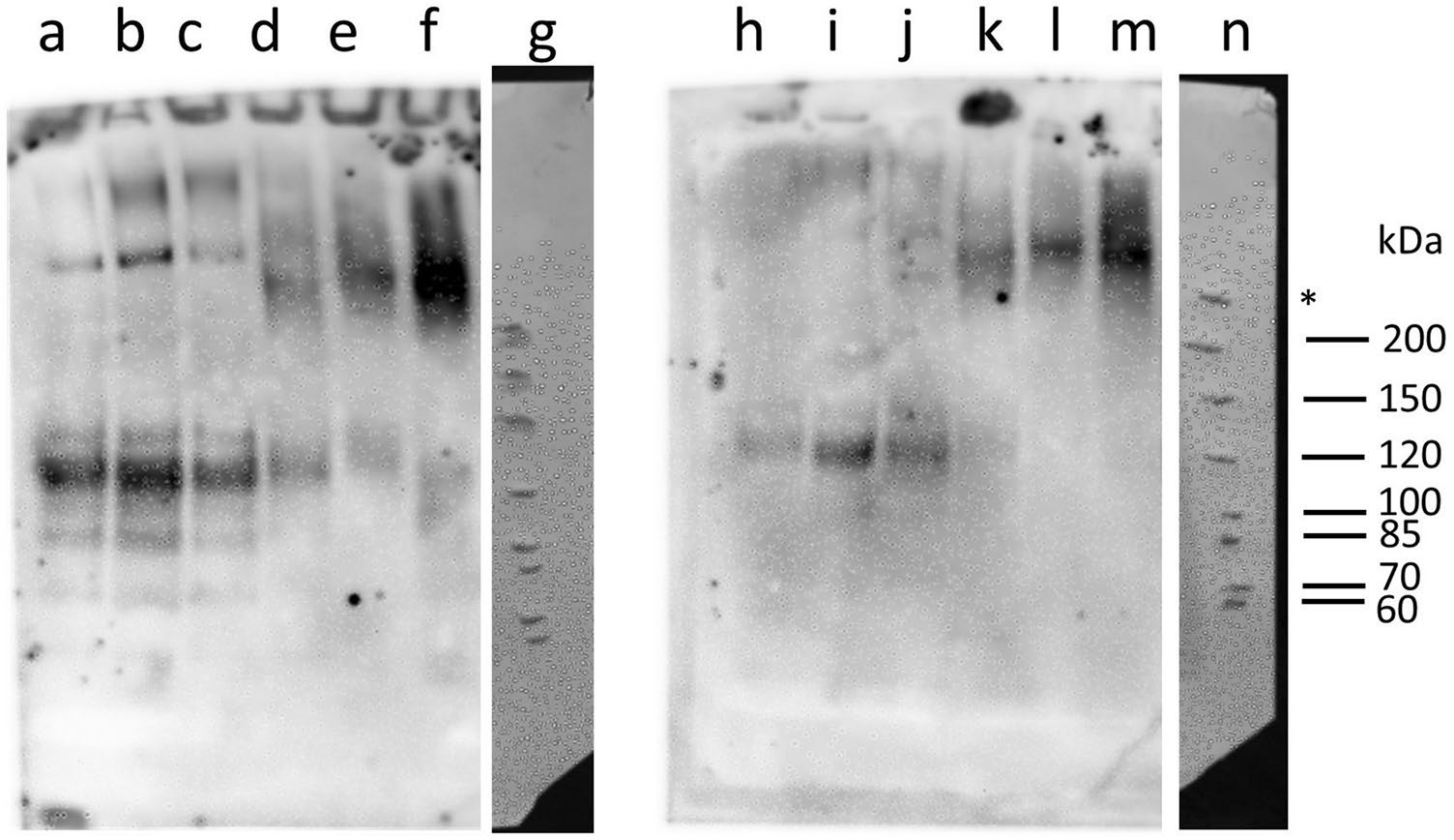
Total and mutant HTT in blood and saliva from premanifest HD individuals. Fifteen micrograms of blood (**a**–**c**,**h**–**j**) and saliva (**d**–**f**,**k**–**m**) were probed with antibodies for total HTT (1:500 4E10; lanes **a**–**f**) and mutant HTT (1:500 MW1; lanes **h**–**m**) on two NuPAGE 3–8% Tris–Acetate Protein Gels, run simultaneously in the same electrophoresis and transfer system. Blood and saliva samples were matched (participant 1: **a**,**d**,**h**,**k**; participant 2: **b**,**e**,**i**,**l**; participant 3: **c**,**f**,**j**,**m**). Lane g and m show white-light images of pencil markings of the PageRuler™ Unstained Protein Ladder, marked after Ponceau S membrane staining; *indicates a high molecular weight marker detected on Ponceau S staining, which does not correspond to any PageRuler™ Unstained Protein Ladder listed molecular weights.

Considering tHTT and mHTT correlations differed between blood and saliva, we hypothesized that these proteins may be uniquely processed in each biofluid. We therefore measured tHTT and mHTT protein levels in saliva and blood from *HTT* mutation-carriers, using Western Blot. This preliminary qualitative investigation of tHTT (Fig. 3, lanes a–f) and mHTT (Fig. 3, lanes h–m) protein in 600 µg/ml aliquots of blood (Fig. 3a–c,h–j) and saliva (Fig. 3d–f,k–m) from 3 PM participants showed distinct bands in each biofluid.

## Discussion

In this study, we have observed significant differences in salivary and plasma mHTT protein levels, but not salivary or plasma tHTT levels, when comparing controls, PM and HD cohorts. Specifically, a significantly greater proportion of PM individuals had detectable salivary and plasma mHTT levels compared to controls. A greater proportion of HD individuals also had detectable salivary mHTT levels compared to controls. The absence of significant different in tHTT in HD compared to controls contrasts with a reported decrease in tHTT in human peripheral blood mononuclear cells, measured by ELISA assay and published by Massai and colleagues^28^, as well as our previously reported increase in salivary tHTT in manifest HD patients compared to controls, also measured by ELISA assay^9^. In our previous study, salivary tHTT was positively correlated with UHDRS score and negatively correlated with TFC^9^, relationships not observed in the current study. However, we did observe significant associations between saliva tHTT levels and other clinical measures: Chorea score, TMS, and DCL. These associations, in addition to correlations between salivary tHTT and mHTT, and salivary mHTT and CAP, PIN and predicted years to manifest onset score in our PM + HD cohort, suggests that these markers are associated with disease progression. Curiously however, while salivary tHTT levels were positively associated with Chorea and TMS in our PM cohort, they were negatively associated in our HD cohort. It is possible that the association in manifest HD patients is influenced by other factors of more severe disease progression. Indeed, differential neurochemical changes have previously been associated with early-disease chorea, compared to late-disease rigidity in HD^29^. In addition, we have previously reported significant differences in levels of plasma neurofilament light between PM and HD cohorts^30^, further suggesting a notable neurophysiological shift associated with disease progression. Based on findings from the current study, salivary tHTT levels may increase with motor symptoms, measured here by Chorea score and TMS, in PM individuals, and subsequently decrease with further motor deterioration, or the onset of rigidity, in manifest HD individuals.

Regarding the discrepancy between our two studies, the cohorts used in the previous study and current study did not differ in demographic or disease characteristics, however the two studies did differ in assay methodology, sensitivity and median HD salivary tHTT levels (ELISA (1500 fM) vs SMC (469fM)). Furthermore, the ELISA utilized in our previous study included an antibody which targets a region between amino acids 802–940^9^, whereas the SMC assay used in the current study targets a proline at amino acid 1220. Previously, an extensive investigation of HTT fragments in a mouse model of HD identified 14 prominent N-terminal fragments ranging from the mutant exon 1 fragment to full length HTT^31^. It is therefore possible that our previous and current studies have documented two different disease pathologies: an increase in full-length tHTT expression^9^, countered by the occurrence of possible protein degradation starting from the 3’ region. Alternatively, it is possible that a high concentration of proteolytic enzymes in saliva, contributed by bacteria and oral cavity cells^20,32^, has lead to substantial degradation of HTT, thereby affecting the observed results. Specifically, such degradation may have influenced our reported findings for salivary tHTT, as a portion of HTT proteins ‘captured’ during the incubation step with the N-terminal 2B7 antibody, may by cleaved in such a way that they lack the epitope for the D7F7 antibody, which binds around amino acid residue 1220^33^. However, previous research has shown that the removal of insoluble material, including bacteria-bound mucins, during saliva sample processing, as well as the the maintenance of samples on ice during handling, and the storage of saliva samples at − 80°C—all of which were performed in this study—preserved the integrity of salivary proteins^32^. Interestingly, Massai and colleagues, who reported a decrease in tHTT in human peripheral blood mononuclear cells, utilized an antibody which targets amino acids 1844–2131 of the HTT protein^28^, which is much closer to the 3′ terminal than those antibodies used in either of our reported studies, suggesting that if degradation is responsible for this discrepancy between studies, it is not unique to saliva. Iit is important to note, however, that the antibody used by Massai and colleagues, 4E10, has also been shown to recognize a 1–573 amino acid N-terminal fragment of HTT in a poly-Q dependent manner, suggesting potential reactivity with one or more additional HTT epitopes^34^. Without the existence of a calibration set of recombinant tHTT proteins of varying fragment lengths, the measure of tHTT protein, which may be cleaved by numerous proteases, remains a relative quantitative assay, and the potential to compare across assay and antibody platforms is limited^9,34,35^.

The absence of inter-biofluid correlations between plasma and salivary tHTT or mHTT, yet the presence of intra-biofluid correlations between saliva tHTT and mHTT, but not plasma tHTT and mHTT, observed in the current study, supports the likelihood that HTT in each biofluid has a different origin, or that there are biofluid-specific variations in transcriptional or post-translational regulation or aggregation. A 2015 analysis of CSF and plasma, using the same single-molecule counting immunoassay as the current study, similarly found no association between mHTT in the two biofluids^7^. The three main salivary glands—the sublingual, submandibular and parotid glands—are innervated by the superior and inferior salivary nuclei, located in the brainstem^36^, which may provide a physiological explanation for the determination of saliva-specific correlations, particularly given that brainstem neurodegeneration has been proposed in HD^37^. Indeed, it is possible that salivary protein levels have a greater direct association with cortical or subcortical pathophysiology, compared to protein changes in blood. It would be of interest to determine whether salivary tHTT and mHTT levels are associated with those in CSF, or entirely unique. We provide further preliminary evidence for biofluid-specific post-translational modification or processing of blood and salivary tHTT and mHTT, through the presentation of unique tHTT and mHTT bands in a western blot analysis of each biofluid. Interestingly, our preliminary western blot findings, using the 4E10 antibody to detect tHTT, show one band above 200 kDa in saliva, compared to the same band and multiple smaller bands in plasma. When assessed using the polyQ-binding MW1 antibody, only the > 200 kDa band is present in saliva, and only the smaller (≤ 120 kDa) bands are present in plasma. This conflicts with our earlier hypothesized scenario in which proteolytic degradation is high in saliva, and suggests greater proteolytic cleavage in plasma. Considered within the context of our earlier scenario in which cleaved 2B7-captured HTT fragments are not detected by the SMC D7F7 antibody, these 120 kDa HTT fragments, detected in plasma with the amino acid 1844–2131 targeting 4E10 antibody, would similarly not be detected by the SMC assay. As aforementioned, the proteolytic cleavage of HTT, and the lack of a calibration set of recombinant tHTT proteins of varying fragment lengths, is a recognized limitation in assessing HTT protein levels.

This study is not without limitations. In addition to the potential presence of proteolytic enzymes in saliva and/or plasma, our limited sample size and the recording of mHTT below the lower limit of quantification in a number of these samples, restricts the conclusions that we can draw from these findings. The detection of plasma and salivary mHTT in our control cohort must also be addressed, however is not unique to our study. Previous studies have detected mHTT in control blood, and cheek buccal cells, and these studies have suggested that assay interfering factors present in the plasma collection tubes, or off-target background detection in the absence of a target, may be the cause of mHTT detection in controls^22,38,39^. In addition, while the MW1 antibody is frequently used and validated in HD research, MW1 binds to a non-pathological glutamine length (Q_10_) and a number of previous studies have suggested that it may bind to wild-type HTT^40–42^. Such lack of specificity may also explain the detection of mHTT in our control cohort. The MW1 antibody is also limited in that it’s binding affinity is affected by CAG repeat length, such that individuals with higher CAG repeat lengths would show greater levels of MW1 binding. We have accounted for the effect of CAG repeat length on MW1 binding by adjusting for this covariate in our correlation analyses, however acknowledge that some variance may still remain.

Our research group is unique in its analysis of HTT protein from whole saliva, and we acknowledge that much work still needs to be done to optimize both the mHTT assay, and the use of this assay in saliva.

Overall, our study presents a compelling argument for the inclusion and analysis of saliva to further the understanding, diagnosis and prognosis of HD. While saliva has been long used as a diagnostic tool for local afflictions which directly affect the oral cavity, such as viral load, drug screening, it has been underappreciated as a vector for CNS-sourced biomarkers of neurodegenerative and psychiatric disorders. We show that mHTT is uniquely processed in saliva, compared to blood, and contend that further investigation into this biofluid will provide new information regarding the development and disease progression of Huntington’s Disease.

## Supplementary Information

Supplementary Information.

## Data Availability

Anonymized summary data are available from the corresponding author by reasonable formal request from qualified researchers, subject to a data sharing agreement and in compliance with the requirements of the funding bodies and institutions.
