# Supplementary material for: Salivary Huntingtin protein is uniquely associated with clinical features of Huntington’s disease
Source: Sci Rep. 2023 Jan 19;13:1034. doi: 10.1038/s41598-023-28019-y (PMC9852574; doi:10.1038/s41598-023-28019-y)
Supplement: Supplementary file 1 — Supplementary Information. [file 41598_2023_28019_MOESM1_ESM.pdf]

Title: Salivary Huntingtin protein is uniquely associated with clinical features of Huntington's Disease

Georgia M Parkin, PhD[1,2]*, Jody Corey-Bloom, MD, PhD[3], Chase Snell, BSc[3], Haileigh Smith, BSc[3], Angela Laurenza[4§] BSc, Manuel Daldin[4] MSc, Alberto Bresciani[4#] MSc, Elizabeth A. Thomas, PhD[1,2]

[1]Department of Epidemiology, University of California Irvine, Irvine, CA, USA;

[2]Institute for Interdisciplinary Salivary Bioscience Research, University of California Irvine, Irvine, CA, USA.

[3]Department of Neurosciences, University of California San Diego, San Diego, CA, USA;

[4]Department of Translational Biology, IRBM S.p.A., via Pontina Km 30, 600, Pomezia, Rome, Italy

[§] Current address: Menarini Ricerche S.p.A.,via Tito Speri 10, Pomezia, Rome, Italy
[#] Current address: Exscientia, Oxford Science Park, Oxford, UK

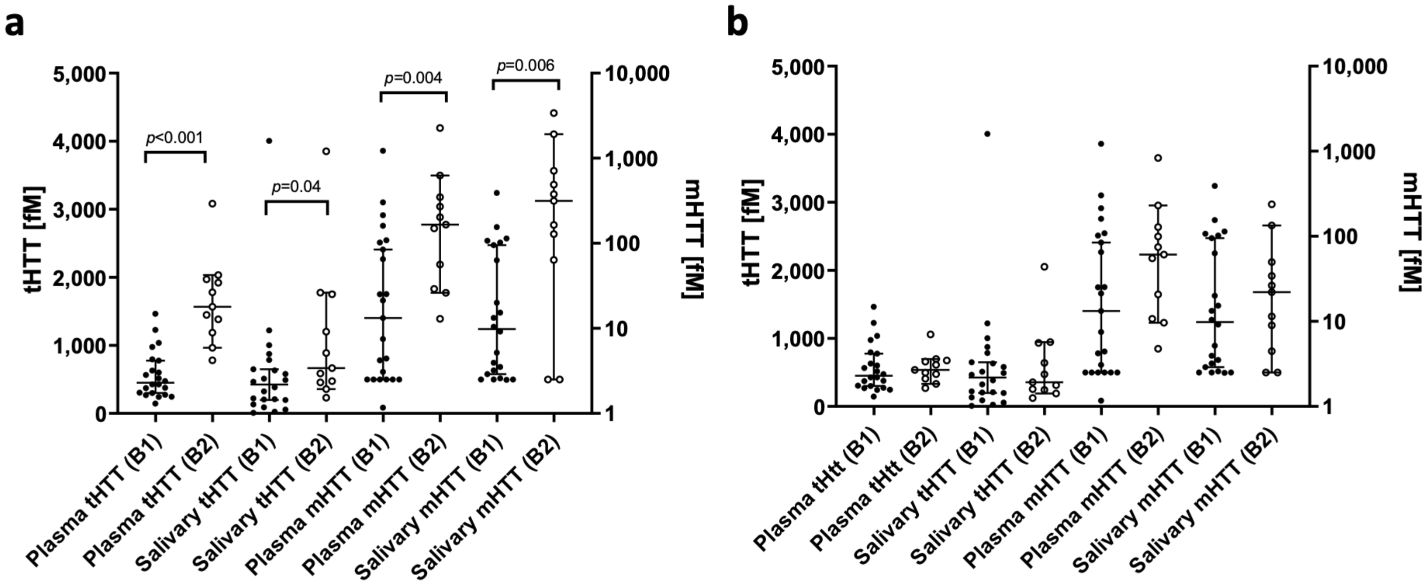

Suppl. Figure 1. HTT analyte batch correction. HTT analyte values in laboratory analysis Batch 1 (B1) and Batch 2 (B2), before (a) and after (b) normalization of Batch 2 by the Batch 1 mean/Batch 2 mean ratio for each analyte. Error bars represent median +/- 95% CI; comparisons conducted using Mann-Whitney U test.

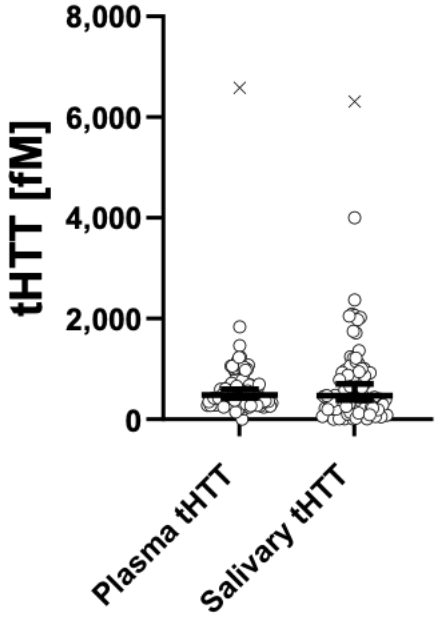

Suppl. Figure 2. **HTT analyte outliers excluded.** Plasma and salivary tHTT values identified as outliers (‘X’ data points) and excluded from analyses. Error bars represent median ± 95% CI.

Suppl. Table 1. Relationship between plasma and salivary tHTT, mHTT, with clinical factors after adjusting for Age, CAG, Sex (r, *p*)

	**PM (n=34)**				**HD (n=19)**				**PM+HD (n=53)**			
	**Plasma tHTT**	**Plasma mHTT**	**Salivary tHTT**	**Salivary mHTT**	**Plasma tHTT**	**Plasma mHTT**	**Salivary tHTT**	**Salivary mHTT**	**Plasma tHTT**	**Plasma mHTT**	**Salivary tHTT**	**Salivary mHTT**
Plasma tHTT		0.22, 0.25	-0.18, 0.34	-0.10, 0.61		-0.19, 0.49	-0.17, 0.56	-0.17, 0.56		0.12, 0.42	-0.13, 0.40	-0.10, 0.53
Plasma mHTT			-0.23, 0.23	-0.11, 0.57			0.30, 0.29	-0.23, 0.42			-0.09, 0.55	-0.23, 0.13
Salivary tHTT				**0.56, <0.001**				0.27, 0.35				**0.51, <0.001**
**Demographic data**												
Education	0.35, 0.06	-0.01, 0.97	-0.01, 0.98	-0.22, 0.25	0.14, 0.62	-0.13, 0.64	0.36, 0.20	0.26, 0.37	0.18, 0.21	-0.02, 0.88	0.17, 0.27	-0.01, 0.95
**Disease data**												
PIN score	0.06, 0.76	0.29, 0.12	0.20, 0.29	0.26, 0.16	-0.12, 0.66	-0.35, 0.19	**-0.58, 0.03**	-0.10, 0.73	-0.14, 0.34	0.04, 0.76	-0.02, 0.92	0.25, 0.10
YTO 60%	0.22, 0.24	0.09, 0.62	-0.20, 0.29	-0.28, 0.13	0.30, 0.26	0.19, 0.48	**0.53, 0.049**	0.11, 0.70	**0.31, 0.03**	0.09, 0.53	-0.12, 0.44	**-0.36, 0.01**
**Clinical Data**												
TFC	-0.09, 0.63	-0.26, 0.15	-0.10, 0.61	-0.10, 0.60	0.39, 0.13	-0.12, 0.66	0.46, 0.10	-0.06, 0.83	0.15, 0.29	-0.18, 0.22	0.03. 0.84	-0.18, 0.23
Independence	-0.29, 0.12	-0.34, 0.06	-0.01, 0.96	-0.16, 0.41	0.19, 0.48	-0.15, 0.59	-0.02, 0.95	-0.25, 0.39	0.01, 0.93	-0.21, 0.14	-0.07, 0.65	-0.21, 0.16
SDMT	-0.16, 0.39	-0.26, 0.15	-0.05, 0.81	-0.02, 0.93	0.15, 0.57	0.28, 0.29	0.30, 0.29	0.01, 0.98	0.08, 0.58	-0.10, 0.49	0.02, 0.88	-0.08, 0.59
MoCA	-0.12, 0.55	0.01, 0.96	0.08, 0.69	-0.02, 0.92	-0.01, 0.97	0.27, 0.30	0.15, 0.61	-0.37, 0.19	-0.10, 0.49	0.04, 0.81	0.12, 0.41	0.02, 0.88
MMSE	0.07, 0.73	0.16, 0.39	-0.06, 0.75	-0.02, 0.92	-0.03, 0.91	0.26, 0.34	0.31, 0.28	-0.02, 0.96	0.02, 0.89	0.13, 0.37	0.06, 0.69	-0.09, 0.57
TMS	-0.12, 0.54	0.20, 0.29	**0.39, 0.03**	**0.50, 0.005**	0.08, 0.77	-0.43, 0.10	**-0.65, 0.01**	-0.14, 0.63	-0.13, 0.36	-0.01, 0.96	0.14, 0.33	**0.36, 0.01**
Chorea	-0.15, 0.44	-0.15, 0.43	**0.47, 0.01**	**0.46, 0.01**	0.16, 0.55	**-0.54, 0.03**	**-0.54, 0.046**	-0.35, 0.22	-0.17, 0.26	-0.19, 0.18	0.16, 0.28	0.26, 0.08

DCL	-0.21, 0.26	0.12, 0.52	**0.44, 0.02**	**0.52, 0.004**					-0.19, 0.20	-0.01, 0.93	0.27, 0.07	**0.39, 0.006**
PBA Total	0.12, 0.51	-0.04, 0.85	0.24, 0.20	0.15, 0.42	-0.33, 0.22	0.32, 0.22	0.43, 0.13	-0.21, 0.48	-0.04, 0.78	0.03, 0.86	**0.36, 0.01**	0.23, 0.15
HADSSIS-Total	0.14, 0.45	-0.06, 0.76	0.16, 0.42	0.04, 0.84	-0.37, 0.16	0.45, 0.08	0.20, 0.50	-0.17, 0.56	-0.08, 0.60	0.08, 0.60	0.24, 0.11	0.12, 0.42
TUG	0.14, 0.57	-0.06, 0.81	0.17, 0.48	0.37, 0.12	0.11, 0.73	-0.09, 0.77	-0.16, 0.62	0.34, 0.28	0.10, 0.57	-0.09, 0.60	0.10, 0.58	**0.43, 0.01**
SWR	-0.14, 0.44	-0.20, 0.26	-0.14, 0.45	**-0.37, 0.03**	0.23, 0.40	0.34, 0.20	0.10, 0.73	-0.12, 0.68	0.01, 0.95	-0.03, 0.83	-0.05, 0.76	-0.19, 0.20
cUHDRS	-0.23 0.20	-0.22, 0.20	-0.21, 0.25	**-0.37, 0.04**	0.22, 0.41	0.22, 0.42	0.40, 0.16	-0.08, 0.80	0.05, 0.74	-0.10, 0.47	-0.04, 0.82	-0.22, 0.13

CAP, CAG and Age Product; YTO 60%, years to predicted manifest onset at 60% probability; TFC, Total Functional Capacity; SDMT, Symbol Digit Modalities Test; MoCA, Montreal Cognitive Assessment; MMSE, Mini-Mental State Examination; TMS, Total Motor Score; DCL, Diagnostic Confidence Interval; PBA Total, Problem Behaviors Assessment Total, HADS-SIS, Hospital Anxiety and Depression Scale-Snaith's Irritability Scale; TUG, Timed Up and Go; cUHDRS, composite Huntington's Disease Rating Scale. Grey cells contained significant correlations ($p<0.05$) before adjusting for covariates (see Table 3).

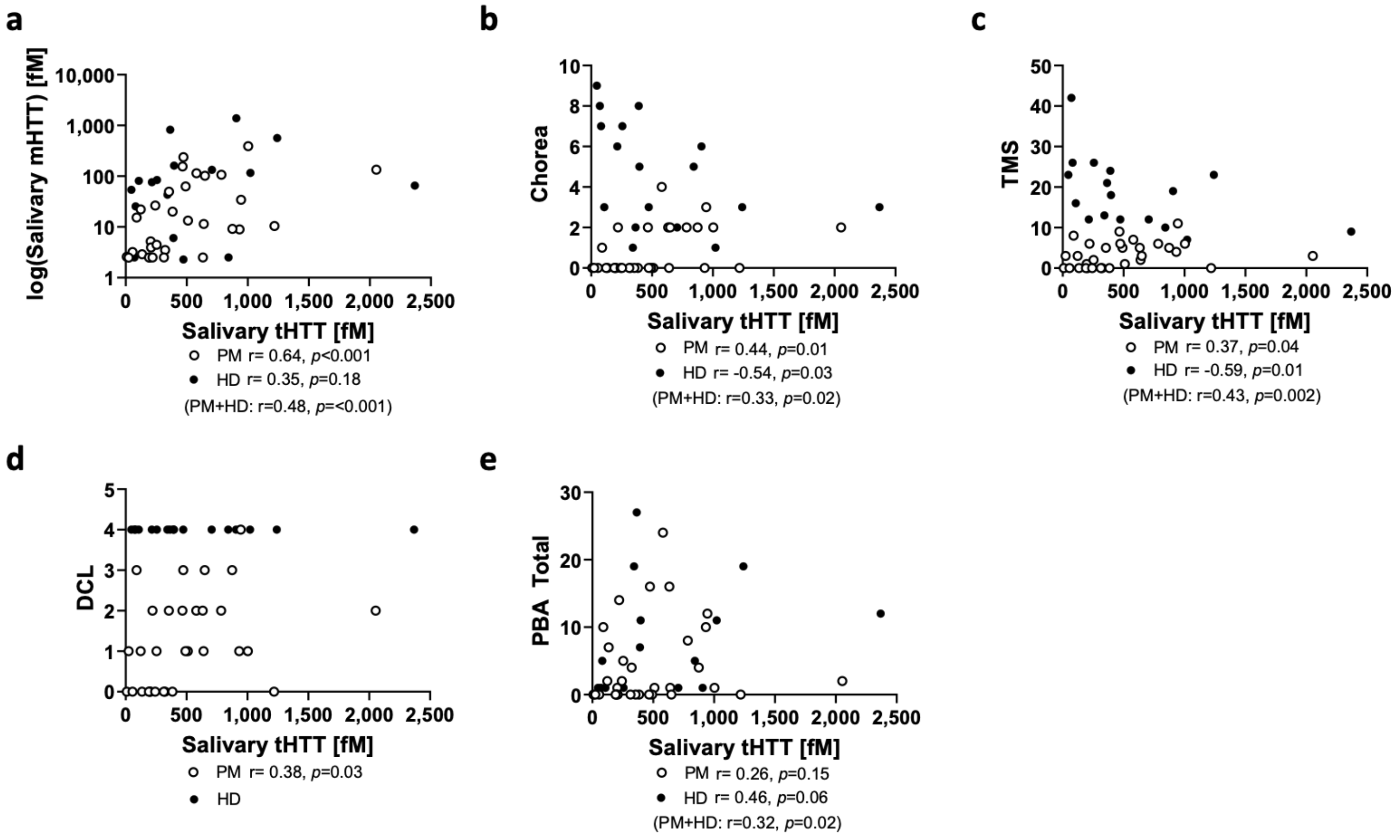

Suppl. Figure 3. Relationship between salivary tHTT and clinical measures. Graphical presentation of significant correlations of saliva tHTT with saliva mHTT (a), Chorea score (b), Total Motor Score (TMS; c), diagnostic confidence level (DCL; d) and Problem Behaviors Assessment Total (PBA Total) score (e), in premanifest (PM; hollow circles) and manifest HD (HD; filled circles) participants. One additional data point excluded visually (saliva tHTT = 4004 fM) to support visual presentation.

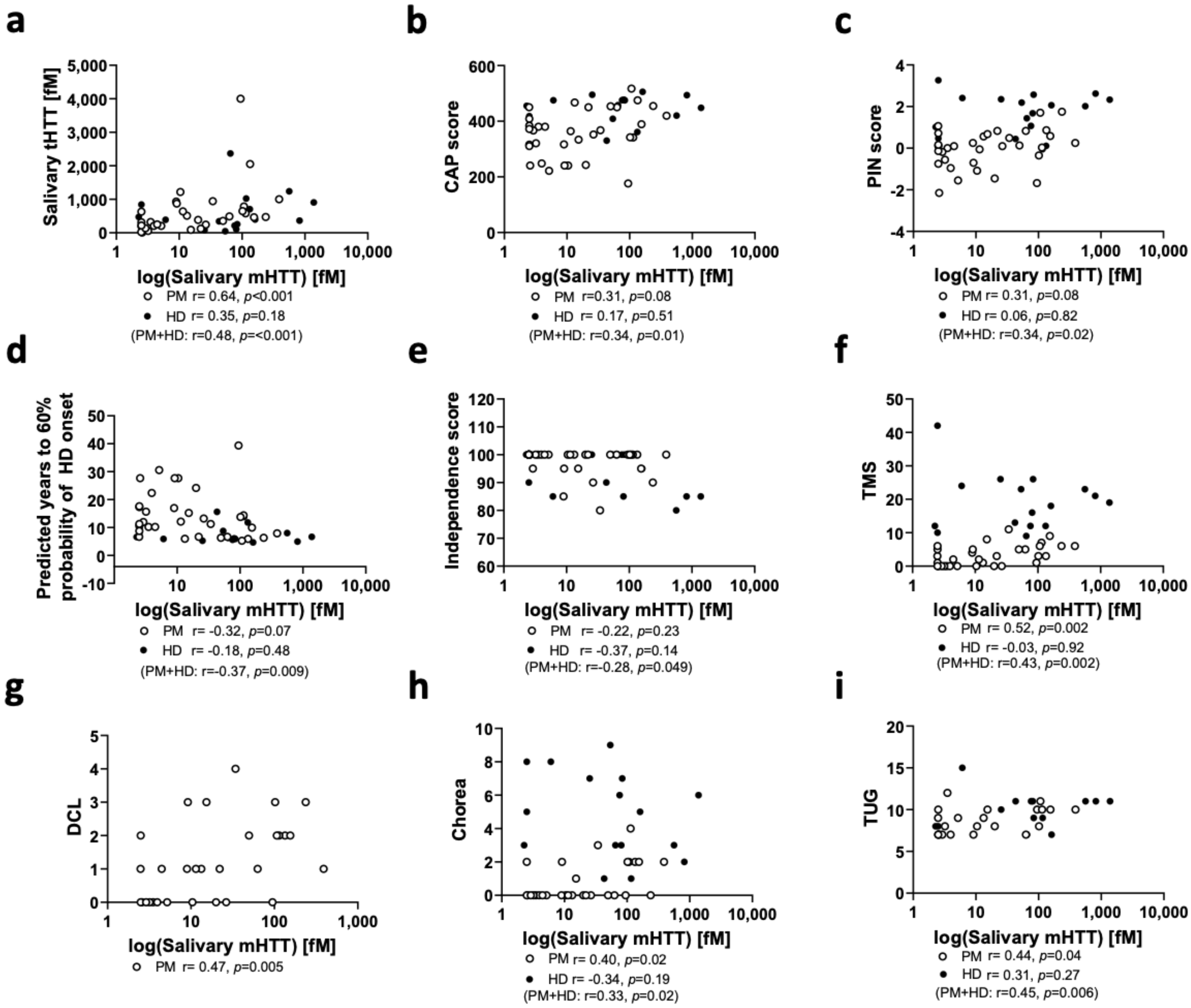

Suppl. Figure 4. Relationship between salivary mHTT and clinical measures. Graphical presentation of significant correlations of salivary mHTT with salivary tHTT (a), CAG-Age Product (CAP) score (b), normalized prognostic index (PIN) score (c), predicted years to 60% probability of manifest symptom onset (d), Independence score (e), Total Motor Score (TMS, f), diagnostic confidence level (DCL, g), Chorea (h), and Timed Up and Go (TUG) score (i), in premanifest (PM; hollow circles) and manifest HD (HD; filled circles) participants.
